# Interpersonal Violence Around Pregnancy Experienced by Rural and Urban Canadian Women: Correlates and Selected Health Outcomes

**DOI:** 10.1177/08862605211043576

**Published:** 2021-09-07

**Authors:** Yingying Su, Carl D’Arcy

**Affiliations:** 1 University of Saskatchewan, Saskatoon, SK, Canada

**Keywords:** interpersonal violence, pregnancy, correlates, health outcomes, urban, rural

## Abstract

Interpersonal violence around pregnancy is of increasing global public health concern affecting both women themselves and their children. The primary aim of this study is to explore and identify potential correlates of such violence and to examine maternal and birth outcomes subsequent to that violence in a nationally representative sample of urban and rural women in Canada. The data are from the Maternity Experiences Survey (MES), a Canadian population-based postcensus survey administered to 6,421 Canadian mothers in 2006. Survey participants were 15 years and older and had given birth to a singleton and continued to live with their infant at the time of the survey. The survey response rate was 78%. Multivariable logistic regression analyses were used in the analysis with adjustments made for confounding variables. The study findings indicated that living in an urban environment was associated with an increased risk of interpersonal violence experience around the time of pregnancy (*OR* = 1.31, 95% CI: 1.03-1.66). In addition, being aboriginal, young, unmarried, economically disadvantaged, a nonimmigrant, and having more than four pregnancies, as well as cigarette smoking, alcohol drinking and drug use before the pregnancy were correlated with interpersonal violence around pregnancy. Maternal interpersonal violence experiences were also associated with postnatal depression and stressful life events among both urban and rural mothers. However, maternal interpersonal violence experiences were only associated with preterm birth among rural mothers but not among urban mothers. The present study highlights the need to implement effective interventions for women experiencing interpersonal violence around pregnancy due to its potential impact on maternal and newborn’s physical and mental health. Screening and intervention should be targeted high-risk women particularly those who are indigenous, young, unmarried, nonimmigrants, of lower socioeconomic status, and manifesting high risk health behaviors.

## Introduction

Abuse and interpersonal violence of women and girls is recognized as a global public health concern associated with morbidity and mortality as well as a violation of women’s rights (Granja et al., 2002). It is reported that approximate one in every five women worldwide suffer different types of abuse and interpersonal violence during lifetime that would give rise to injury or even death (World Health Organization, 2005). Abuse and interpersonal violence (including physical or sexual abuse) during pregnancy and the postpartum period has many serious clinical adverse consequences for both mother, fetus, and child and far-reaching societal implications (Kingston et al., 2016). Pregnancy can be a stressful and anxious time. It is a vulnerable period when physical, emotional, and social change occurs (Van Parys et al., 2014). If pregnant women experience abuse and interpersonal violence around pregnancy, either physical or emotional, it can have a detrimental effect for both themselves and their offspring (Campbell & Lewandowski, 1997). A growing body of literature reports that pregnant women who suffered interpersonal violence often present with multiple risk factors that may increase the risk of physical damage and perinatal mental health adversity for mothers and child. These adversities include increased risk of preterm birth (PB), low birth weight (LBW) or mortality for newborns (Bailey, 2010; Beydoun et al., 2012; Howard et al., 2013; Janssen et al., 2012; Urquia et al., 2011). For the mothers themselves there is an increased risk of chronic diseases, sexually transmitted diseases, and post-traumatic stress disorder, etc. (Coker et al., 2004; Curry et al., 1998). In addition, the literature reports numerous risk factors or correlates of abuse and interpersonal violence around the time of pregnancy. For example, young age, lower educational status, single marital status, aboriginal ancestry, substance use, smoking and alcohol use prior to pregnancy (Brownridge et al., 2011; Kingston et al., 2016; Taillieu & Brownridge, 2010).

Abuse and interpersonal violence against pregnant women persists in both developed and developing countries (Nasir & Hyder, 2003). From the research in North America and Europe, the prevalence of abuse and violence suffered by pregnant women varies between 0.9% and 22.0%. The variation in prevalence reported in these studies may be due to sampled populations and different study materials, methodologies, and cultural context differences across study sites and countries (Finnbogadottir et al., 2014; Hedin et al., 1999; James et al., 2013; Stenson et al., 2001). Physical abuse is reported as the most frequent type of abuse and it may lead to adverse pregnancy outcomes, at the same time, it is a modifiable risk factor for adverse pregnancy outcomes (Gazmararian et al., 1996; Rodrigues et al., 2008).

There has been much theorizing about differences in abuse and interpersonal violence across rural and urban areas. Social theorists have long discussed the effects of the urbanization and industrialization in terms of the transformation of social relationships, social norms, and culture. The changes are seen as altering the density, intensity, and nature of social relationships. Modern social capital theory stresses *structural* features such as organizational membership and cognitive elements such as trust, reciprocity and mutual help to characterize differences among communities. These diverse social structural features of communities are seen to impact on both the interpersonal violence prevalence and its related health outcomes of rural and urban inhabitants.

Some studies have reported that rural areas have lower levels of education, more socioeconomic deprivation, and geographical remoteness, leading to a higher prevalence of abuse and interpersonal violence being experienced by pregnant women (Bhandari et al., 2015; Goins et al., 2005; Pong et al., 2011; Tiwari et al., 2008). Furthermore, the unavailability in specialist care in rural areas, which may have an additional negative effect on the interpersonal violence experiences of pregnant women in rural areas. A Nigerian cross-sectional study found that residing in a rural area increased the risk of violence around the time of pregnancy (Tella et al., 2020). However, a prospective longitudinal study recruiting participants from three U.S. urban clinics found that the prevalence of physical abuse during pregnancy is higher in urban mothers with low-income, with approximately one in five women reported such experiences during pregnancies (Alhusen et al., 2013). In contrast a narrative review of 63 studies indicates that rates of intimate partner violence are generally similar across rural, urban, and suburban locales (Edwards, 2015). Although a survey from the South America generally found higher levels of domestic violence among urban women compared with rural women (Van Dis et al., 2002). Similarly, it has been reported that living in urban areas was statistically significantly associated with violence exposure during pregnancy among African women (Rurangirwa et al., 2017). Likewise, Van Horne (2010) found intimate partner violence was related to population density with higher levels occurring in more densely populated counties.

Although it is important to understand the contextual influences of interpersonal violence around pregnancy in rural and urban areas, our understanding of how rural and urban areas differ is limited and most of potential practice and policy implications were developed for rural areas alone (Bhandari et al., 2011; Singh et al., 2018). A better understanding of the attributes and consequences of the abuse and interpersonal violence experiences for both urban and rural mothers is an important direction for future strategies implemented at the individual, provider, and community level. In addition, it is important to broaden the reach of prenatal/postnatal care to vulnerable groups who may actually benefit. These targeted efforts on the particular challenges faced by vulnerable mothers are crucial to the success of efforts to reduce severe maternal and birth outcomes.

Canada has a very large geographical area but with a relatively small population that is largely spread out. There are social structural inequalities between rural and urban areas in Canada and differences have also been identified between rural areas bordering on urban hubs and more remote rural areas. Such inequalities include economic structures, social reproduction and socioeconomic axes (e.g., race and ethnicity, educational attainment) (Pampalon et al., 2010). We are not aware of any Canadian research that has explored interpersonal violence toward women during pregnancy in rural and urban areas. Our proposed study will fill this knowledge gap.

In the present study, we explored the prevalence of interpersonal violence against pregnant women in rural and urban areas of Canada. The correlates associated with the experience of interpersonal violence and related maternal and birth outcomes among rural and urban mothers were also examined.

## Methods

### Study Subjects

The Maternity Experiences Survey (MES) was the first and only national survey devoted to pregnancy, labor, birth, and postpartum experiences in Canada. The survey was conducted by Statistics Canada and sponsored by the Public Health Agency of Canadian Perinatal Surveillance System. It is a population-based postcensus survey conducted between October 23, 2006, and January 31, 2007. The Canadian Census of 2006 was used to define the target population of women, who had given birth between 15th February and 15th May 2006 (for the provinces) and 1st November 2005 and 1st February 2006 (for the territories), were 15+ years of age at the baby’s birth, whose baby was born in Canada and lived with the mother at least one night per month since then. Mothers living on First National reserves and in collective dwellings were excluded. An estimated 76,500 women residents in Canada met these criteria. This targeted sample frame was stratified by residence, mother’s age, other children in the household, with mothers less than 20 years of age being over sampled. A simple random sample was selected without replacement within each stratum. The sample targeted 8,542 women. In total, 6,421 of them responded to the survey yielding response rate of 78%. Computer-assisted telephone interviewing (CATI) technology was used combined with a personal interview with a paper version of the questionnaire where a telephone interview was not possible. Response to the survey was voluntary and all participants provided informed consent.

The MES data is made available to bona fide researchers by Statistics Canada through the MES Master File, which does not contain any personal identifiers. Statistics Canada provided survey weights for researchers to use in estimating population parameters. The MES Master File was accessed at the Saskatchewan Research Data Centre (SKY-RDC), a joint Statistics Canada—University of Saskatchewan data portal.

### Measures

#### Demographic characteristics and correlates.

Standard Statistics Canada questions were asked concerning maternal age at birth of reference baby, maternal education, marital status, total household income, aboriginal ancestry, location of residence, province or territory of residence (data not shown in the table), immigration status, sex of reference baby, maternal age at their first pregnancy, total number of pregnancies, smoking status before pregnancy, alcohol consumption before pregnancy, and drug use before pregnancy.

The variables were categorized as follows: *participants’ age at birth of survey reference baby—*<20 years, 20-29 years, 30-39 years, and 40+ years; *maternal age at first pregnancy—*<20 years, 20-34 years, and 35+ years; *maternal education—*university graduation and above, postsecondary diploma, some postsecondary education, high school graduation, and less than high school; *marital status—*married/common-law, divorced/separated/widow and single; *total household income* (in Canadian dollars circa 2006)—$100,000 or more, $60,000-$100,000, $30,000-$60,000, $10,000-$30,000, and less than $10,000; *residence location—*rural (<1,000 inhabitants or population density < 400/km^2^) versus urban; *total number of pregnancies—*1, 2-3, ≥4.

#### Interpersonal violence.

Ten questions adapted from the Violence Against Women Survey were used to assess the acts of physical or sexual violence experience of Canadian women around the time of pregnancy (Statistics Canada, 1993). Participants were asked whether a spouse or partner or anyone else has done any of the following things to them in the last two years: (1) threatened to hit you with his or her fist or anything else that could have hurt you; (2) thrown anything at you that could have hurt you; (3) pushed, grabbed or shoved you in a way that could have hurt you; (4) slapped you; (5) kicked you, bit you or hit you with his or her fist; (6) hit you with something that could have hurt you; (7) beaten you; (8) choked you; (9) used or threatened to use a gun or knife on you; and (10) forced you into any unwanted sexual activity by threatening you, holding you down, or hurting you in some way. Cronbach’s alpha was 0.98.

The above 10 items were categorized into four categories as *Any abuse and interpersonal violence experience—*an affirmative answer to one or more of the 10 items; *Violence, threats, or potential hurting acts—*at least one affirmative answer to questions 1-3; *Physical violence—*at least one affirmative answer to questions 4-9; *Sexual violence—*an affirmative answer to question 10. Abuse victims were also asked about the *frequency* of these incidents happened in the past two years with responses ranging from 1 to more than 11 times. The response of women to the question about their relationship to the *violence perpetrator* was categorized as: husband or boyfriend, a family member, a friend or acquaintance, and a stranger and other person.

#### Interpersonal violence related outcomes.

The interpersonal violence related outcomes assessed were postnatal depression, stressful events, PB, LBW, and type of delivery. *Depression* was measured using the Edinburgh Postnatal Depression Scale (EPDS) (Cox et al., 1987). The scale consisted of 10 questions with four response categories scored from 0 to 3, a cutoff score of 11 or more is used to indicate, with reasonable sensitivity and specificity, a high probability of having postpartum depression (Smith-Nielsen et al., 2018). Cronbach’s alpha value was 0.69. *Stressful life events—*using a modified Newton and Hunt Stressful Life Events Scale (Newton & Hunt, 1984), respondents were asked about the occurrence of 13 stressful life events during the 12 months prior to giving birth. Cronbach’s alpha was 0.64. *LBW* was defined as less than 2,500 grams at birth. *PB* was defined as a delivery before 37 completed gestational weeks. *Type of delivery* was categorized as caesarean or vaginal.

### Statistical Analysis

To account for the complex sampling design, Statistics Canada survey sample weights and bootstrapping procedures were used in the statistical analyses.

Descriptive statistics were used to summarize perpetrators of violence, violence times, violence types, and violence around the time of pregnancy in rural and urban locales. The chi-square test was used to determine the difference in sociodemographic characteristics between the urban and rural groups. The relationships between location of residence and interpersonal violence were analyzed with univariable and multivariable logistic regression. Three models were constructed while controlling for different types of potential confounders and effect modifiers. In addition, multivariable logistic regression analyses were then used to further investigate maternal and newborn outcomes of interpersonal violence with adjustments for a variety of intervening variables. Odds ratios (ORs) and their 95% confidence intervals (95%CIs) were calculated to indicate the strength of the association. Stata, version 9.0, was used for the analyses.

## Results

Table 1 provides the basic characteristics of rural and urban mothers in the MES survey sample. All differences are statistically significant except for drug use before the pregnancy. In *rural areas*, more than half of (51.5%) the mothers were 20-29 years of age at the birth of the survey reference child, most were married (92.7%) and had completed high school (91.2%), 9.6% were immigrants, and more than half (52.8%) had a household income of less than $60,000 income annually. Approximately half the mothers (51.5%) had two to three pregnancies. In *urban areas*, approximately half of (49.0%) participants were 30-39 years of age at the birth of survey reference child and 91.5% were married or living common-law. In total, 24.4% mothers were immigrants and one third (39.8%) were highly educated (university graduation and above). Similarly, a large proportion of mothers’ (50.5%) household income was less than $60,000 per year and less than half (49.7%) had 2-3 pregnancies. The proportion of mothers living in rural areas reporting interpersonal violence around the time of pregnancy was 9.8% (95% CI = 9.3%-10.3%) whereas for mothers living in urban areas the prevalence was 11.1% (95% CI = 10.9%-11.4%).

**Table 1.** Basic Characteristics of the Rural and Urban Mothers in the Maternal Experience Survey (MES).

Variables		Rural (Weighted *n* = 13,108)		Urban (Weighted *n* = 63,400)		χ^2^	*p* Value
		Frequency	%	Frequency	%		
Sex of the baby	Male	7,042	53.7%	32,526	51.3%	25.5	**.000**
	Female	6,066	46.3%	30,874	48.7%		
Maternal age at birth (years)	15-19	392	3.0%	1,870	2.9%	198.3	**.000**
	20-29	6,754	51.5%	28,521	45.0%		
	30-39	5,647	43.1%	31,068	49.0%		
	Above 40	315	2.4%	1,941	3.0%		
Immigrant status	Yes	1,258	9.6%	15,500	24.4%	1393.0	**.000**
	No	11,850	90.4%	47,900	75.6%		
Marital status	Married/common-law	12,151	92.7%	57,983	91.5%	24.3	**.000**
	Divorced/separated/widow	234	1.8%	1,204	1.9%		
	Single	723	5.5%	4,213	6.6%		
Mother’s education	Less than high school	1,153	8.8%	4,633	7.3%	552.0	.000
	High school graduation	2,023	15.4%	8,026	12.7%		
	Some postsecondary education	984	7.5%	3,626	5.7%		
	Postsecondary diploma	5,143	39.2%	21,890	34.5%		
	University graduation and above	3,805	29.0%	25,224	39.8%		
Household income	Less than $10,000	176	1.3%	1,435	2.3%	219.1	**.000**
	$10,000 to less than $30,000	1,879	14.3%	8,797	13.9%		
	$30,000 to less than $60,000	4,878	37.2%	21,784	34.3%		
	$60,000 to less than $100,000	4,071	31.1%	19,043	30.0%		
	$100,000 or more	2,104	16.1%	12,341	19.5%		
Maternal age at first pregnancy (years)	15-19	2,564	19.6%	10,402	16.4%	126.6	**.000**
	20-34	10,164	77.5%	50,176	79.1%		
	Above 35	380	2.9%	2,821	4.5%		
Total number of pregnancies	1	3,909	29.8%	21,801	34.4%	115.4	**.000**
	2 to 3	6,802	51.9%	31,514	49.7%		
	≥4	2,397	18.3%	10,085	15.9%		
Aboriginal ancestry	Yes	695	5.3%	2,529	4.0%	46.4	**.000**
	No	12,413	94.7%	60,871	96.0%		
Abuse around the time of pregnancy	Yes	1,286	9.8%	7,060	11.1%	19.6	**.000**
	No	11,823	90.2%	56,339	88.9%		
Smoked before this pregnancy	Yes	3,445	26.3%	13,377	21.1%	182.2	**.000**
	No	9,663	73.7%	50,022	78.9%		
Alcohol consumption before pregnancy	Yes	8,516	65.0%	39,079	61.6%	203.4	**.000**
	No	4,592	35.0%	24,321	38.4%		
Drug use before this pregnancy	Yes	929	7.1%	4,216	6.7%	3.3	.069
The result in bold is significant at p < .05.	No	12,179	92.9%	59,183	93.3%		

*Note.* The result in bold is significant at p < .05.

**Figure 1.**
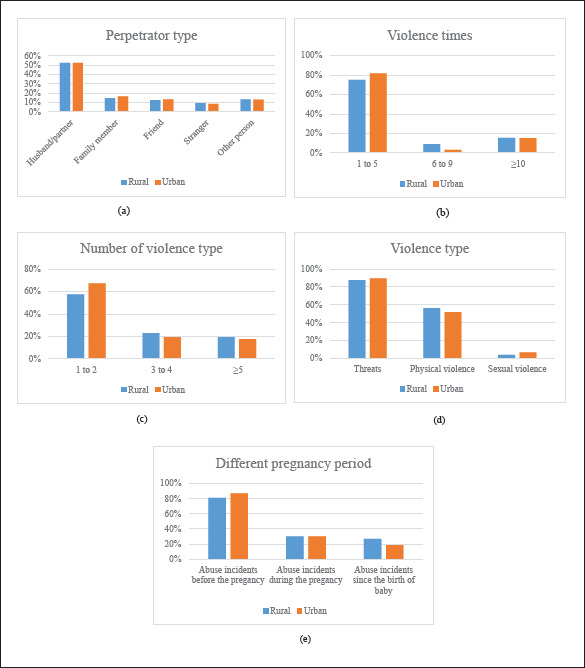
Violence characteristics in rural and urban areas.

The percentage of mothers reporting 1-5 instances of interpersonal violence around the time of pregnancy, was 75.2% in rural areas and 81.7% in urban areas. A husband or partner was the most common perpetrator of interpersonal violence accounting for 52.2% and 52.4% of perpetrators in rural and urban areas, respectively. The prevalence of interpersonal violence by a family member, friend, stranger, or other perpetrator was significantly lower in both areas. More than half of abused mothers in rural and urban areas had experienced one to two different types of interpersonal violence (57.5% and 64.5%, respectively). Threats or potential hurting acts were the most common type of interpersonal violence, with sexual violence being the least frequent type of abuse and interpersonal violence among both rural and rural mothers. Mothers who experienced physical or sexual violence also usually experienced threats or hurting acts as well. The patterns of interpersonal violence experienced before pregnancy, during pregnancy, and after childbirth were similar for both rural and urban mothers. Violence incidents were less frequent during the pregnancy than before the pregnancy and dropped dramatically after the child’s birth. Among rural mothers who had experienced violence around the time of pregnancy, 81.1% reported violence incidents before the pregnancy, 30.4% reported incidents during the pregnancy and 26.9% reported incidents after childbirth; and the same percentages for urban mothers were 87.0%, 30.4%, and 18.7%. Figure 1 provides details.

In the unadjusted analyses, there was no difference in the correlates for interpersonal violence experience around the time of pregnancy between mothers living in rural and urban areas. However, after adjusting for maternal education, household income, marital status, aboriginal ancestry, province/territory of residence, sex of baby, immigration status, mother’s age at birth of survey reference child, mother’s age at first pregnancy, and total number of pregnancies, urban mothers were at 1.32 times increased odds of experiencing interpersonal violence around pregnancy compared to rural mothers (*OR* = 1.32, 95% CI 1.05-1.67, *p* = .02). In the final multivariable logistic regression model with full adjustment (Table 2), this association was slightly attenuated but still significant indicating that living in an urban environment was associated with an increased risk of experiencing interpersonal violence around the time of pregnancy (*OR* = 1.31, 95% CI: 1.03-1.66, *p* = .03). In addition, mothers who were young (*OR* = 2.19, 95% CI: 1.51-3.18, *p* < .01), single or divorced (*OR*_single_ = 2.70, 95% CI: 2.04-3.56, *p* < .01; *OR*_divorced_ = 2.87, 95% CI: 1.74-4.73, *p* < .01), with low income (*OR* = 1.99, 95% CI: 1.13-3.51, *p* < .01), a nonimmigrant (*OR* = 1.62, 95% CI: 1.17-2.27, *p* < .01), of aboriginal ancestry (*OR* = 1.48, 95% CI: 1.04-2.09, *p* = .01), and had more than four pregnancies (*OR* = 1.60, 95% CI: 1.17-2.19, *p* < .01) were more likely to experience interpersonal violence. In addition, cigarette smoking (*OR* = 1.65, 95% CI: 1.34-2.03, *p* < .01), alcohol consumption (*OR* = 1.30, 95% CI: 1.05-1.62, *p* = .04), and drug use (*OR* = 2.01, 95% CI: 1.53-2.64, *p* < .01) before the pregnancy were significantly associated with an increased risk of interpersonal violence around pregnancy. Details are presented in the Table 2.

**Table 2.** Association Between Residency and Interpersonal Violence Around the Time of Pregnancy.

Variables		Model 1		Model 2		Model 3	
Demographic							
Area size	Rural	1		1		1	
	Urban	1.15	0.93,1.43	**1.32**	**1.05, 1.67**	**1.31**	**1.03, 1.66**
Mother age at birth (years)	15-19			**2.03**	**1.40, 2.94**	**2.19**	**1.51, 3.18**
	20-29			1		1	
	30-39			**0.68**	**0.53, 0.87**	**0.70**	**0.54, 0.90**
	Above 40			0.65	0.34, 1.26	0.69	0.35, 1.35
Mother’s education	University graduation and above			1		1	
	Postsecondary diploma			1.06	0.83, 1.34	0.98	0.76, 1.25
	Some postsecondary education			**1.71**	**1.20, 2.44**	1.42	0.99, 2.03
	High school graduation			1.25	0.94, 1.68	1.06	0.78, 1.44
	Less than high school			1.34	0.95, 1.89	1.12	0.78, 1.61
Household income	$100,000 or more			1		1	
	$60,000 to less than $100,000			0.99	0.72, 1.36	0.98	0.71, 1.34
	$30,000 to less than $60,000			1.11	0.81, 1.53	1.04	0.75, 1.45
	$10,000 to less than $30,000			**1.85**	**1.30, 2.64**	**1.61**	**1.12, 2.30**
	Less than $10,000			**2.15**	**1.22, 3.78**	**1.99**	**1.13, 3.51**
Marital status	Married/common-law			1		1	
	Divorced/Separated/Widow			**2.98**	**1.82, 4.89**	**2.87**	**1.74, 4.73**
	Single			**3.12**	**2.39, 4.08**	**2.70**	**2.04, 3.56**
Aboriginal ancestry	No			1		1	
	Yes			**1.71**	**1.22, 2.38**	**1.48**	**1.04, 2.09**
Immigrant status	Yes			1		1	
	No			**2.18**	**1.60, 2.98**	**1.62**	**1.17, 2.27**
Obstetric factors							
Sex of the survey reference baby	Male			1		1	
	Female			1.01	0.85, 1.21	1.04	0.87, 1.25
Mother’s age at first pregnancy (years)	15 to 19			1		1	
	20-34			**0.49**	**0.38, 0.63**	**0.54**	**0.42, 0.70**
	≥35			**0.50**	**0.26, 0.98**	0.56	0.29, 1.11
Total number of pregnancies	1			1		1	
	2 to 3			1.14	0.92, 1.42	1.20	0.96,1.50
	≥4			**1.51**	**1.12, 2.05**	**1.60**	**1.17, 2.19**
Health behaviors							
Smoked before this pregnancy	Yes					**1.65**	**1.34, 2.03**
	No					1	
Alcohol consumption before this pregnancy	Yes					**1.30**	**1.05, 1.62**
	No					1	
Drug use before this pregnancy	Yes					**2.01**	**1.53, 2.64**
	No					1	

*Note.* The result in bold is significant at p < .05.

Table 3 shows the outcomes associated with pregnancy related interpersonal violence in this sample of mothers and their neonates. Among *all* Canadian mothers, interpersonal violence experience around the time of pregnancy was significantly associated with postnatal depression (*OR* = 2.51, 95% CI: 2.05-3.09, *p* < .01). After adjusting for confounding factors, the significant association remained (*OR* = 2.35, 95% CI: 1.86-2.97, *p* < .01). Likewise, a history of violence and abuse was significantly associated with the experience of stressful life events (*OR* = 3.82, 95% CI: 3.06-4.77, *p* < .01). This association was attenuated but still remained significant after adjusting for confounders (*OR* = 2.64, 95% CI: 2.09-3.33, *p* < .01). No association was found between interpersonal violence around pregnancy and adverse birth outcomes, including PB, caesarean delivery, and LBW baby.

**Table 3.** Impact of Interpersonal Violence Around the Time of Pregnancy Among Rural and Urban Mothers.

Variables	Rural Area		Urban Area		Overall Sample	
	Nonadjusted Model	Fully Adjusted Model	Nonadjusted Model	Fully Adjusted Model	Nonadjusted Model	Fully Adjusted Model
Postnatal depression						
No	1	1	1	1	1	1
Yes	**2.36 (1.45, 3.81)**	**1.92 (1.17, 3.18)**	**2.53 (2.02, 3.18)**	**2.50 (1.92, 3.25)**	**2.51 (2.05, 3.09)**	**2.35 (1.86, 2.97)**
Stressful events						
No	1	1	1	1	1	1
Yes	**3.62 (2.20, 5.95)**	**2.45 (1.44, 4.17)**	**3.86 (3.01, 4.94)**	**2.66 (2.05, 3.45)**	**3.82 (3.06, 4.77)**	**2.64 (2.09, 3.33)**
Type of delivery						
Vaginal	1	1	1	1	1	1
Caesarean	**0.49 (0.30, 0.82)**	0.56 (0.33, 1.00)	0.94 (0.76, 1.16)	1.05 (0.83, 1.33)	0.86 (0.71, 1.04)	0.96 (0.77, 1.18)
Birth Weight of the baby, g						
<2,500	1	1	1	1	1	1
≥2,500	0.72 (0.29, 1.76)	0.65 (0.26, 1.64)	1.22 (0.78, 1.91)	1.27 (0.79, 2.04)	1.13 (0.75, 1.69)	1.14 (0.75, 1.74)
Gestational age, weeks						
≥37	1	1	1	1	1	1
<37	**2.15 (1.10, 4.19)**	**2.30 (1.09, 4.88)**	0.93 (0.62, 1.39)	0.82 (0.53, 1.27)	1.10 (0.78, 1.55)	0.99 (0.68, 1.43)

*Note.* The result in bold is significant at p < .05.

In a rural-urban stratified analysis, mothers who experienced interpersonal violence were at a greater risk of postnatal depression in the nonadjusted model for both rural and urban areas (*OR*_rural_ = 2.36, 95% CI: 1.45-3.81, *p* < .01; *OR*_urban_ = 2.53, 95% CI: 2.02-3.18, *p* < .01). After adjustment for potential intervening variables the rural and urban differences were attenuated to 1.92 times increased odds (*OR* = 1.92, 95% CI 1.17-3.18, *p* = .01) and 2.50 times increased odds (*OR* = 2.50, 95% CI 11.92-3.25, *p* < .01), respectively. Similar patterns were observed for stressful events. In the unadjusted models, interpersonal violence in the past two years was associated with increased frequency of stressful events in rural (*OR* = 3.62, 95% CI: 2.20-5.95, *p* < .01) and urban areas (*OR* = 3.86, 95% CI: 3.01-4.94, *p* < .01). After further adjustments for potential confounders, the experience of interpersonal violence was still associated with stressful events among rural (*OR* = 2.45, 95% CI: 1.44-4.17, *p* < .01) and urban mothers (*OR* = 2.66, 95% CI: 2.05-3.45, *p* < .01). In contrast there were no instances of LBW among the offspring of abused mothers in comparison with nonabused mothers in either rural or urban environments. However, the experience of interpersonal violence was associated with an increased risk of a PB among rural mothers only, but not among urban mothers, in both unadjusted and fully adjusted models (*OR*_unadjusted_ = 2.15, 95% CI 1.10-4.19, *p* = .03; *OR*_adjusted_ = 2.30, 95% CI 1.09-4.88, *p* = .03).

## Discussion

This study explored abuse and interpersonal violence around the time of pregnancy among rural and urban mothers in Canada identifying potential correlates and examining related health outcomes.

Our study findings indicated that in comparison with rural mothers, urban mothers may experience somewhat higher levels of abuse and interpersonal violence around the time of pregnancy. Interpersonal violence across all three time periods around pregnancy was more common among mothers of aboriginal ancestry, nonimmigrant, and unmarried mothers who resided in an urban area, had a low income, who had more than four pregnancies and who had a history of smoking/drinking/drug use before the pregnancy. This experience of interpersonal violence was significantly correlated with postnatal depression, stressful events in both residential areas, but was only related to preterm delivery of the baby among rural mothers.

Our findings differ from previous reports that found rural women reporting a higher risk of abuse and interpersonal violence experience (Bueno & Lopes, 2018; Shannon et al., 2006) who theorized that rural mothers usually have inadequate awareness of their rights and limited access to health services (Dimah & Dimah, 2004). However, the current study’s finding of slightly higher levels of violence in the urban areas may be due to competing resources for urban mothers which may make it less feasible for them to leave the abusive environment and rebuild their life, thus prolonging their dependency on abusive partners and making them more vulnerable to abuse and violence (Bhandari et al., 2015). Our finding showed that both urban and rural mothers experienced more violence incidents before the pregnancy and less violence incidents after the baby’s birth which is consistent with previous studies (Beydoun et al., 2010). The present study also found similar patterns of perpetrator characteristics, number of violent incidents, and types of violence in urban and rural areas.

Besides residency, other socioeconomic characteristics including being young and unmarried were found to be significantly associated with interpersonal violence around pregnancy. Our findings are in line with a USA National Crime Victimization Survey demonstrating that violence and abuse around the time of pregnancy is more likely to occur in women who are young, and separated or divorced (Rennison, 2001). In addition, Brown et al. (2008) found that young maternal age was an important factor linked to the interpersonal violence experienced by pregnant women. Devries et al. (2010) found that young maternal age at childbirth may represent a more general socioeconomically disadvantaged characteristic leading to a higher risk of intimate partner violence. Also consistent with our study findings, a meta-analysis and systematic review with 55 independent studies reported that being unmarried and of lower socioeconomic status were associated with abuse and violence around the time of pregnancy (James et al., 2013). These relationships have also been confirmed in several studies (Heaman, 2005).

In agreement with our finding results, being of aboriginal ancestry has been associated with an increased risk of spousal abuse and interpersonal violence in several other Canadian studies (Nelson et al., 2018; Nihaya Daoud et al., 2013). Aboriginal women in abusive relationships may face unique challenges when seeking to change their abusive environment due to contextual factors such as differences in community social resources and/or services (Blagg et al., 2018; Nihaya Daoud et al., 2013). Colonization theory suggests that contextual factors related to colonialism could account for increased interpersonal violence and abuse around the time of pregnancy experienced by Aboriginal mothers (Daoud et al., 2013).

Likewise, compared to Canadian-born mothers, immigrant mothers consistently report experiencing less abuse and interpersonal violence around pregnancy which is consistent with our study findings (Khanlou et al., 2017). Immigration from other countries to Canada are mostly economic migrants, hence they are more likely to be equipped with skills and have a higher level of education. However, it has been noted that interpersonal violence may be underreported among immigrant women. A lack of knowledge and access to social services, financial dependence on the partner, and fears of deportation may discourage immigrant women from reporting violent interpersonal incidents (Du Mont & Forte, 2012).

It is not surprising that women who had more pregnancies were more likely to report the abuse and violence. The violence often starts or gets worse during the beginning of pregnancy. In addition, more pregnancies increase the chance of unintended pregnancies. Women with unintended pregnancies have been found to have a greater risk of abuse and interpersonal violence during pregnancy (Lukasse et al., 2015).

In line with our study results, several behavioral lifestyle factors have been found to be linked to violence around pregnancy, for instances, alcohol consumption before pregnancy (Nihaya Daoud et al., 2013; Stöckl et al., 2010), smoking before pregnancy (Chu et al., 2010) and drug use before pregnancy (Campbell, 2002).

Many studies have found associations between the experience of violence during pregnancy and negative maternal and neonatal outcomes (Alhusen et al., 2015). The findings of the current study are generally consistent with those that described significant links between experiencing interpersonal violence and stressful events as well as postnatal depression with stronger associations being found among urban mothers. The results of our study are comparable with prior literature showing an association between interpersonal violence and negative health behaviors among mothers residing in both rural and urban areas (Bailey & Daugherty, 2007; Melville et al., 2010; Small et al., 2008). It is suggested that these relationships are stronger for urban populations because urban women have a greater tendency toward economic and emotional dependency (Sigalla et al., 2017). However, an increased risk of PB was only found among rural mothers who had experiences of interpersonal violence around the time of pregnancy. Direct and indirect mechanisms for how violence during pregnancy may influence adverse maternal and neonatal outcomes have been proposed (Coker et al., 2004). A direct causal path between physical abuse and PB may occur through blows to the abdomen or sexual assault. Indirect mechanisms are through elevated stress-related hormones, such as levels of corticotrophin-releasing hormone (CRH). Higher levels of HPA hormones could initiate labor as well as restrict uteroplacental perfusion (Kalantaridou et al., 2010).

*Strengths and limitations.* There were several limitations in the present study. First, due to the nature of cross-sectional study design, our ability to draw causal inferences is limited and our findings should be interpreted only as correlations. Second, women with a history of abuse and interpersonal violence may be less likely to take part in the survey or be reluctant to disclose such experiences which would result in the underestimation of the prevalence of interpersonal violence. Third, mothers who lived on First Nations Reserves representing socially and economically disadvantaged populations were not surveyed in the MES, thereby reducing generalizability of the results. However, Aboriginal mothers living off reserve as well as mothers with aboriginal ancestry have been included in the MES. Fourth, data on emotional abuse, which appears to be the most prevalent form of abuse and interpersonal violence, was not collected in the MES though thus the present study has not captured the full scope of the abuse and interpersonal violence behaviors.

Even with these limitations in mind, the present study has several strengths. First of all, although the MES data were collected in 2006, it is the first and only national population-based Canadian survey of the experiences of interpersonal violence around the time of pregnancy at the national level across all provinces. This study reinforces previous work that demonstrates a range of negative health consequences for women who experience violence during pregnancy and includes compromised child health outcomes. Other studies of abuse and violence around pregnancy have been largely conducted in health care or social services settings thereby limiting the generalizability of study findings to the broader population context. Furthermore, the present study considered a variety of attributes across various domains, mitigating the effects of confounders. Reducing abuse and interpersonal violence around pregnancy could be achieved effectively by identifying women at potentially higher risk and providing support to reduce their social and economic disadvantages, and offering health care providers opportunities to intervene. Bystander intervention and structured community responses to interpersonal violence in both rural and urban areas should be enhanced.

## Conclusions

Given that interpersonal violence exposures lead to adverse consequences for the women themselves and their child’s birth outcomes as well as their child’s future physical and emotional development, identification of mothers at potentially greater risk, with counseling support and referral to appropriate agencies with effective programs should be encouraged. The developmental origins of health and disease hypothesis stresses the fundamental role of early life experiences on future physical and emotional development. Reducing women’s risk of violence and the potential negative sequelae of that violence for mother and child should be a priority. Our study also highlights the need for universal violence screening and referral services for pregnant women keying in on interpersonal violence. It is crucial to provide victims of abuse and violence with nonjudgmental, sensitive, and supportive care services as early as possible during their pregnancies and to reach out to vulnerable women in not only rural areas but urban areas to prevent further episodes of violence.

## Author Biographies

**Yingying Su**, MSc, is a PhD candidate in School of Public Health at the University of Saskatchewan, Canada. Her thesis examines the biopsychosocial model of stress: risk factors, mediating effects, moderating effects and impacts.

**Carl D’Arcy**, PhD, is a professor of Department of Psychiatry and School of Public Health at the University of Saskatchewan. He has broad research interests primarily concerned with the cause, course, outcome, and the treatment/prevention of common psychiatric and neurodegenerative disorders-mood and anxiety disorder, substance abuse disorders, Alzheimer’s disease, and dementia as well as psychological well-being.

## References

[bibr1-08862605211043576] Alhusen J. L., Lucea M. B., Bullock L., & Sharps P. (2013). Intimate partner violence, substance use, and adverse neonatal outcomes among urban women. *The Journal of Pediatrics*, 163(2), 471-476. 23485028 10.1016/j.jpeds.2013.01.036 PMC3686908

[bibr2-08862605211043576] Alhusen J. L., Ray E., Sharps P., & Bullock L. (2015). Intimate partner violence during pregnancy: Maternal and neonatal outcomes. *Journal of Women’s Health*, 24(1), 100-106. 10.1089/jwh.2014.4872 PMC4361157 25265285

[bibr3-08862605211043576] Bailey B. A. (2010). Partner violence during pregnancy: Prevalence, effects, screening, and management. *International Journal of Women’s Health*, 2(1), 183-197. 10.2147/ijwh.s8632 PMC2971723 21072311

[bibr4-08862605211043576] Bailey B. A., & Daugherty R. A. (2007). Intimate partner violence during pregnancy: Incidence and associated health behaviors in a rural population. *Maternal and Child Health Journal*, 11(5), 495-503. 17323125 10.1007/s10995-007-0191-6

[bibr5-08862605211043576] Beydoun H. A., Al-Sahab B., Beydoun M. A., & Tamim H. (2010). Intimate partner violence as a risk factor for postpartum depression among Canadian women in the maternity experience survey. *Annals of Epidemiology*, 20(8), 575-583. 20609336 10.1016/j.annepidem.2010.05.011 PMC4179881

[bibr6-08862605211043576] Beydoun H. A., Beydoun M. A., Kaufman J. S., Lo B., & Zonderman A. B. (2012). Intimate partner violence against adult women and its association with major depressive disorder, depressive symptoms and postpartum depression: A systematic review and meta-analysis. *Social Science & Medicine*, 75(6), 959-975. 22694991 10.1016/j.socscimed.2012.04.025 PMC3537499

[bibr7-08862605211043576] Bhandari S., Bullock L. F., Anderson K. M., Danis F. S., & Sharps P. W. (2011). Pregnancy and intimate partner violence: How do rural, low-income women cope? *Health Care for Women International*, 32(9), 833-854. 21834721 10.1080/07399332.2011.585532 PMC4432839

[bibr8-08862605211043576] Bhandari S., Bullock L. F., Richardson J. W., Kimeto P., Campbell J. C., & Sharps P. W. (2015). Comparison of abuse experiences of rural and urban African American women during perinatal period. *Journal of Interpersonal Violence*, 30(12), 2087-2108. 25315478 10.1177/0886260514552274 PMC4682574

[bibr9-08862605211043576] Blagg H., Williams E., Cummings E., Hovane V., Torres M., & Woodley K. N. (2018). *Innovative models in addressing violence against Indigenous women: Final report*. ANROWS, Horizons, 01.

[bibr10-08862605211043576] Brown S. J., McDonald E. A., & Krastev A. H. (2008). Fear of an intimate partner and women’s health in early pregnancy: Findings from the maternal health study. *Birth*, 35(4), 293-302. 19036042 10.1111/j.1523-536X.2008.00256.x

[bibr11-08862605211043576] Brownridge D. A., Taillieu T. L., Tyler K. A., Tiwari A., Chan K. L., & Santos S. C. (2011). Pregnancy and intimate partner violence: Risk factors, severity, and health effects. *Violence Against Women*, 17(7), 858-881. 21775311 10.1177/1077801211412547

[bibr12-08862605211043576] Bueno A. L., & Lopes M. J. (2018). Rural women and violence: Readings of a reality that approaches fiction. *Ambiente & Sociedade*, 21. https://www.scielo.br/j/asoc/a/VVNcs38qHFGC5q3yvh8xPzj/

[bibr13-08862605211043576] Campbell J. C. (2002). Health consequences of intimate partner violence. *The Lancet*, 359(9314), 1331-1336. 10.1016/S0140-6736(02)08336-8 11965295

[bibr14-08862605211043576] Campbell J. C., & Lewandowski L. A. (1997). Mental and physical health effects of intimate partner violence on women and children. *Psychiatric Clinics of North America*, 20(2), 353-374. 9196919 10.1016/s0193-953x(05)70317-8

[bibr15-08862605211043576] Chu S. Y., Goodwin M. M., & D’Angelo D. V. (2010). Physical violence against U.S. women around the time of pregnancy, 2004-2007. *American Journal of Preventive Medicine*, 38(3), 317-322. 20171534 10.1016/j.amepre.2009.11.013

[bibr16-08862605211043576] Coker A. L., Sanderson M., & Dong B. (2004). Partner violence during pregnancy and risk of adverse pregnancy outcomes. *Paediatric and Perinatal Epidemiology*, 18(4), 260-269. 15255879 10.1111/j.1365-3016.2004.00569.x

[bibr17-08862605211043576] Cox J. L., Holden J. M., & Sagovsky R. (1987). Detection of postnatal depression: Development of the 10-item Edinburgh Postnatal Depression Scale. *The British Journal of Psychiatry*, 150(6), 782-786. 3651732 10.1192/bjp.150.6.782

[bibr18-08862605211043576] Curry M. A., Perrin N., & Wall E. (1998). Effects of abuse on maternal complications and birth weight in adult and adolescent women. *Obstetrics & Gynecology*, 92(4 Pt. 1), 530-534. 9764624 10.1016/s0029-7844(98)00258-0

[bibr19-08862605211043576] Daoud N., Smylie J., Urquia M., Allan B., & O’Campo P. (2013). The contribution of socio-economic position to the excesses of violence and intimate partner violence among Aboriginal versus non-Aboriginal women in Canada. *Canadian Journal of Public Health*, 104(4), e278-e283. 24044466 10.17269/cjph.104.3724 PMC6973901

[bibr20-08862605211043576] Devries K. M., Kishor S., Johnson H., Stöckl H., Bacchus L. J., Garcia-Moreno C., & Watts C. (2010). Intimate partner violence during pregnancy: Analysis of prevalence data from 19 countries. *Reproductive Health Matters*, 18(36), 158-170. 21111360 10.1016/S0968-8080(10)36533-5

[bibr21-08862605211043576] Dimah K. P., & Dimah A. (2004). Elder abuse and neglect among rural and urban women. *Journal of Elder Abuse & Neglect*, 15(1), 75-93.

[bibr22-08862605211043576] Du Mont J., & Forte T. (2012). An exploratory study on the consequences and contextual factors of intimate partner violence among immigrant and Canadian-born women. *BMJ Open*, 2(6), Article e001728. 10.1136/bmjopen-2012-001728 PMC3533032 23148344

[bibr23-08862605211043576] Edwards K. M. (2015). Intimate partner violence and the rural–urban–suburban divide: Myth or reality? A critical review of the literature. *Trauma, Violence, & Abuse*, 16(3), 359-373. 10.1177/1524838014557289 25477015

[bibr24-08862605211043576] Finnbogadottir H., Dykes A. K., & Wann-Hansson C. (2014). Prevalence of domestic violence during pregnancy and related risk factors: A cross-sectional study in southern Sweden. *BMC Womens Health*, 14, Article 63. 10.1186/1472-6874-14-63 PMC4030020 24885532

[bibr25-08862605211043576] Gazmararian J. A., Lazorick S., Spitz A. M., Ballard T. J., Saltzman L. E., & Marks J. S. (1996). Prevalence of violence against pregnant women. *JAMA*, 275(24), 1915-1920. 8648873

[bibr26-08862605211043576] Goins R. T., Williams K. A., Carter M. W., Spencer S. M., & Solovieva T. (2005). Perceived barriers to health care access among rural older adults: A qualitative study. *Journal of Rural Health*, 21(3), 206-213. 10.1111/j.1748-0361.2005.tb00084.x 16092293

[bibr27-08862605211043576] Granja A. C., Zacarias E., & Bergstrom S. (2002). Violent deaths: The hidden face of maternal mortality. *BJOG: An International Journal of Obstetrics and Gynaecology*, 109(1), 5-8. 11843374

[bibr28-08862605211043576] Heaman M. I. (2005). Relationships between physical abuse during pregnancy and risk factors for preterm birth among women in Manitoba. *Journal of Obstetric, Gynecologic, & Neonatal Nursing*, 34(6), 721-731. 10.1177/0884217505281906 16282230

[bibr29-08862605211043576] Hedin L. W., Grimstad H., Moller A., Schei B., & Janson P. O. (1999). Prevalence of physical and sexual abuse before and during pregnancy among Swedish couples. *Acta Obstetricia et Gynecologica Scandinavica*, 78(4), 310-315. 10203298

[bibr30-08862605211043576] Howard L. M., Oram S., Galley H., Trevillion K., & Feder G. (2013). Domestic violence and perinatal mental disorders: A systematic review and meta-analysis. *PLoS Medicine*, 10(5), Article e1001452. 10.1371/journal.pmed.1001452 PMC3665851 23723741

[bibr31-08862605211043576] James L., Brody D., & Hamilton Z. (2013). Risk factors for domestic violence during pregnancy: A meta-analytic review. *Violence and Victims*, 28(3), 359-380. 23862304 10.1891/0886-6708.vv-d-12-00034

[bibr32-08862605211043576] Janssen P. A., Heaman M. I., Urquia M. L., O'Campo P. J., & Thiessen K. R. (2012). Risk factors for postpartum depression among abused and nonabused women. *American Journal of Obstetrics and Gynecology*, 207(6), 489.e1-489.e8. 10.1016/j.ajog.2012.09.022 23063016

[bibr33-08862605211043576] Kalantaridou S., Zoumakis E., Makrigiannakis A., Lavasidis L., Vrekoussis T., & Chrousos G. (2010). Corticotropin-releasing hormone, stress and human reproduction: An update. *Journal of Reproductive Immunology*, 85(1), 33-39. 20412987 10.1016/j.jri.2010.02.005

[bibr34-08862605211043576] Khanlou N., Haque N., Skinner A., Mantini A., & Kurtz Landy C. (2017). Scoping review on maternal health among immigrant and refugee women in Canada: Prenatal, intrapartum, and postnatal care. *Journal of Pregnancy*, 2017, Article 8783294. 10.1155/2017/8783294 PMC5292182 28210508

[bibr35-08862605211043576] Kingston D., Heaman M., Urquia M., O'Campo P., Janssen P., Thiessen K., & Smylie J. (2016). Correlates of abuse around the time of pregnancy: Results from a national survey of Canadian women. *Maternal and Child Health Journal*, 20(4), 778-789. 26694044 10.1007/s10995-015-1908-6

[bibr36-08862605211043576] Lukasse M., Laanpere M., Karro H., Kristjansdottir H., Schroll A. M., Van Parys A. S., Wangel A. M., Schei B., & group. Bidens study (2015). Pregnancy intendedness and the association with physical, sexual and emotional abuse—A European multi-country cross-sectional study. *BMC Pregnancy Childbirth*, 15, Article 120. 10.1186/s12884-015-0558-4 PMC4494794 26008119

[bibr37-08862605211043576] Melville J. L., Gavin A., Guo Y., Fan M.-Y., & Katon W. J. (2010). Depressive disorders during pregnancy: Prevalence and risk factors in a large urban sample. *Obstetrics and Gynecology*, 116(5), 1064-1070. 20966690 10.1097/AOG.0b013e3181f60b0a PMC3068619

[bibr38-08862605211043576] Nasir K., & Hyder A. A. (2003). Violence against pregnant women in developing countries: Review of evidence. *European Journal Public Health*, 13(2), 105-107. 10.1093/eurpub/13.2.105 12803407

[bibr39-08862605211043576] Nelson C., Lawford K. M., Otterman V., & Darling E. K. (2018). Original quantitative research mental health indicators among pregnant Aboriginal women in Canada: Results from the maternity experiences survey. *Health Promotion & Chronic Disease Prevention in Canada: Research, Policy & Practice*, 38(7-8), 269-276. 30129714 10.24095/hpcdp.38.7/8.01 PMC6126563

[bibr40-08862605211043576] Newton R. W., & Hunt L. P. (1984). Psychosocial stress in pregnancy and its relation to low birth weight. *British Medical Journal (Clinical Research Ed.)*, 288(6425), 1191-1194. 6424783 10.1136/bmj.288.6425.1191 PMC1441330

[bibr41-08862605211043576] Pampalon R., Hamel D., & Gamache P. (2010). Health inequalities in urban and rural Canada: Comparing inequalities in survival according to an individual and area-based deprivation index. *Health & Place*, 16(2), 416-420. 20022551 10.1016/j.healthplace.2009.11.012

[bibr42-08862605211043576] Pong R. W., DesMeules M., Heng D., Lagace C., Guernsey J. R., Kazanjian A., Manuel D., Pitblado J. R., Bollman R., Koren I., Dressler M. P., Wang F., & Luo W. (2011). Patterns of health services utilization in rural Canada. *Chronic Diseases and Injuries in Canada*, 31(Suppl. 1), 1-36. 22047772

[bibr43-08862605211043576] Rennison C. M. (2001). *Intimate partner violence and age of victim, 1993-99*. US Department of Justice, Office of Justice Programs, Bureau of Justice Statistics. https://bjs.ojp.gov/content/pub/pdf/ipva99.pdf

[bibr44-08862605211043576] Rodrigues T., Rocha L., & Barros H. (2008). Physical abuse during pregnancy and preterm delivery. *American Journal of Obstetrics and Gynecology*, 198(2), 171.e1-171.e6. 10.1016/j.ajog.2007.05.015 17905171

[bibr45-08862605211043576] Rurangirwa A. A., Mogren I., Ntaganira J., & Krantz G. (2017). Intimate partner violence among pregnant women in Rwanda, its associated risk factors and relationship to ANC services attendance: A population-based study. *BMJ open*, 7(2), Article e013155. 10.1136/bmjopen-2016-013155 PMC5337709 28399509

[bibr46-08862605211043576] Shannon L., Logan T., Cole J., & Medley K. (2006). Help-seeking and coping strategies for intimate partner violence in rural and urban women. *Violence and Victims*, 21(2), 167-181. 16642737 10.1891/vivi.21.2.167

[bibr47-08862605211043576] Sigalla G. N., Rasch V., Gammeltoft T., Meyrowitsch D. W., Rogathi J., Manongi R., & Mushi D. (2017). Social support and intimate partner violence during pregnancy among women attending antenatal care in Moshi Municipality, Northern Tanzania. *BMC Public Health*, 17(1), Article 240. 10.1186/s12889-017-4157-3 PMC5343555 28274220

[bibr48-08862605211043576] Singh J. K., Evans-Lacko S., Acharya D., Kadel R., & Gautam S. (2018). Intimate partner violence during pregnancy and use of antenatal care among rural women in southern Terai of Nepal. *Women and Birth*, 31(2), 96-102. 28844866 10.1016/j.wombi.2017.07.009

[bibr49-08862605211043576] Small M. J., Gupta J., Frederic R., Joseph G., Theodore M., & Kershaw T. (2008). Intimate partner and nonpartner violence against pregnant women in rural Haiti. *International Journal of Gynecology & Obstetrics*, 102(3), 226-231. 18675418 10.1016/j.ijgo.2008.05.008 PMC3901698

[bibr50-08862605211043576] Smith-Nielsen J., Matthey S., Lange T., & Væver M. S. (2018). Validation of the Edinburgh Postnatal Depression Scale against both DSM-5 and ICD-10 diagnostic criteria for depression. *BMC Psychiatry*, 18(1), 1-12. 30572867 10.1186/s12888-018-1965-7 PMC6302501

[bibr51-08862605211043576] Statistics Canada. (1993). *Violence against women survey*. https://www23.statcan.gc.ca/imdb/p2SV.pl?Function=getSurvey&SDDS=3896 12295372

[bibr52-08862605211043576] Stenson K., Heimer G., Lundh C., Nordstrom M. L., Saarinen H., & Wenker A. (2001). The prevalence of violence investigated in a pregnant population in Sweden. *Journal of Psychosomatic Obstetrics & Gynecology*, 22(4), 189-197. 11840572 10.3109/01674820109049973

[bibr53-08862605211043576] Stöckl H., Watts C., & Kilonzo Mbwambo J. K. (2010). Physical violence by a partner during pregnancy in Tanzania: Prevalence and risk factors. *Reproductive Health Matters*, 18(36), 171-180. 21111361 10.1016/S0968-8080(10)36525-6

[bibr54-08862605211043576] Taillieu T. L., & Brownridge D. A. (2010). Violence against pregnant women: Prevalence, patterns, risk factors, theories, and directions for future research. *Aggression and Violent Behavior*, 15(1), 14-35.

[bibr55-08862605211043576] Tella A. O., Tobin-West C. I., & Babatunde S. (2020). Experience of domestic violence among pregnant women in rural and urban areas of Niger Delta region of Nigeria: Risk factors, help-seeking resources and coping strategies. *Annals of Ibadan Postgraduate Medicine*, 18(1), 65-73. 33623496 PMC7893296

[bibr56-08862605211043576] Tiwari A., Chan K. L., Fong D., Leung W. C., Brownridge D. A., Lam H., Wong B., Lam C. M., Chau F., Chan A., Cheung K. B., & Ho P. C. (2008). The impact of psychological abuse by an intimate partner on the mental health of pregnant women. *BJOG: An International Journal of Obstetrics and Gynaecology*, 115(3), 377-384. 18190375 10.1111/j.1471-0528.2007.01593.x PMC2253706

[bibr57-08862605211043576] Urquia M. L., O’Campo P. J., Heaman M. I., Janssen P. A., & Thiessen K. R. (2011). Experiences of violence before and during pregnancy and adverse pregnancy outcomes: An analysis of the Canadian maternity experiences survey. *BMC Pregnancy Childbirth*, 11(1), Article 42. 10.1186/1471-2393-11-42 PMC3141595 21649909

[bibr58-08862605211043576] Van Dis J., Mahmoodian M., Goddik S., & Dimitrievich E. (2002). A survey of the prevalence of domestic violence in rural and Urban South Dakota. *South Dakota Journal of Medicine*, 55(4), 133-139. 11977866

[bibr59-08862605211043576] Van Horne S. L. (2010). *The importance of place: A national examination of the structural correlates of intimate partner homicides*. Rutgers University-Graduate School.

[bibr60-08862605211043576] Van Parys A. S., Deschepper E., Michielsen K., Temmerman M., & Verstraelen H. (2014). Prevalence and evolution of intimate partner violence before and during pregnancy: A cross-sectional study. *BMC Pregnancy Childbirth*, 14, Article 294. 10.1186/1471-2393-14-294 PMC4159505 25169813

[bibr61-08862605211043576] World Health Organization. (2005). *Addressing violence against women and achieving the millennium development goals*. https://apps.who.int/iris/bitstream/handle/10665/43361/WHO_FCH_GWH_05.1.pdf;sequence=1

